# In Silico Investigation of AKT2 Gene and Protein Abnormalities Reveals Potential Association with Insulin Resistance and Type 2 Diabetes

**DOI:** 10.3390/cimb45090471

**Published:** 2023-09-12

**Authors:** M. E. Elangeeb, Imadeldin Elfaki, M. A. Elkhalifa, Khalid M. Adam, A. O. Alameen, Ahmed Kamaleldin Elfadl, Ibrahim Altedlawi Albalawi, Kholoud S. Almasoudi, Reema Almotairi, Basim S. O. Alsaedi, Marwan H. Alhelali, Mohammad Muzaffar Mir, Dnyanesh Amle, Rashid Mir

**Affiliations:** 1 Department of Basic Medical Sciences, College of Applied Medical Sciences, University of Bisha, Bisha 61922, Saudi Arabia; 2 Department of Biochemistry, Faculty of Science, University of Tabuk, Tabuk 47512, Saudi Arabia; elfakiimadeldin@gmail.com; 3 Department of Anatomy, Faculty of Medicine and Health Sciences, University of Bisha, Bisha 61922, Saudi Arabia; emelkhalifa@ub.edu.sa; 4 Department of Medical Laboratory Sciences, College of Applied Medical Sciences, University of Bisha, Bisha 61922, Saudi Arabia; dr.aboalbasher@gmail.com; 5 Department of Biomedical Science, Faculty of Veterinary Medicine, King Faisal University, Alahssa 31982, Saudi Arabia; ahmedomer151@hotmail.com; 6 Veterinary Research Section, Ministry of Municipality, Doha P.O. Box 35081, Qatar; ahmedpath@hotmail.com; 7 Department of Pathology, Faculty of Veterinary Medicine, University of Khartoum, Khartoum 11115, Sudan; 8 Department of Surgical Oncology, Faculty of Medicine, University of Tabuk, Tabuk 47512, Saudi Arabia; drbalawi@yahoo.com; 9 Department of Medical Lab Technology, Prince Fahad Bin Sultan Chair for Biomedical Research, Faculty of Applied Medical Sciences, University of Tabuk, Tabuk 71491, Saudi Arabia; kalmasoudi@ut.edu.sa (K.S.A.); ralmotairi@ut.edu.sa (R.A.); 10 Department of Statistics, University of Tabuk, Tabuk 47512, Saudi Arabia; balsaedi@ut.edu.sa (B.S.O.A.); malhilaly@ut.edu.sa (M.H.A.); 11 Department of Basic Medical Sciences, College of Medicine, University of Bisha, Bisha 61922, Saudi Arabia; mmmir@ub.edu.sa; 12 Department of Biochemistry, All India Institute of Medical Sciences, Nagpur 441108, India; dnyaneshamle@gmail.com

**Keywords:** bioinformatics, nsSNP, protein, AKT2, type 2 diabetes, insulin resistance, insulin signaling pathway

## Abstract

Type 2 diabetes (T2D) develops from insulin resistance (IR) and the dysfunction of pancreatic beta cells. The AKT2 protein is very important for the protein signaling pathway, and the non-synonymous SNP (nsSNPs) in AKT2 gene may be associated with T2D. nsSNPs can result in alterations in protein stability, enzymatic activity, or binding specificity. The objective of this study was to investigate the effect of nsSNPs on the AKT2 protein structure and function that may result in the induction of IR and T2D. The study identified 20 variants that were considered to be the most deleterious based on a range of analytical tools included (SIFT, PolyPhen2, Mut-pred, SNAP2, PANTHER, PhD-SNP, SNP&Go, MUpro, Cosurf, and I-Mut). Two mutations, p.A179T and p.L183Q, were selected for further investigation based on their location within the protein as determined by PyMol. The results indicated that mutations, p.A179T and p.L183Q alter the protein stability and functional characteristics, which could potentially affect its function. In order to conduct a more in-depth analysis of these effects, a molecular dynamics simulation was performed for wildtype AKT2 and the two mutants (p.A179T and p.L183Q). The simulation evaluated various parameters, including temperature, pressure, density, RMSD, RMSF, SASA, and Region, over a period of 100 ps. According to the simulation results, the wildtype AKT2 protein demonstrated higher stability in comparison to the mutant variants. The mutations p.A179T and p.L183Q were found to cause a reduction in both protein stability and functionality. These findings underscore the significance of the effects of nsSNPs (mutations p.A179T and p.L183Q) on the structure and function of AKT2 that may lead to IR and T2D. Nevertheless, they require further verifications in future protein functional, protein–protein interaction, and large-scale case–control studies. When verified, these results will help in the identification and stratification of individuals who are at risk of IR and T2D for the purpose of prevention and treatment.

## 1. Introduction

Diabetes mellitus (DM) is a metabolic disease characterized by elevated blood sugar. The general types of DM include type 1 DM, which is caused by the absence of insulin due to pancreatic beta cell destruction mediated by autoimmune mechanisms [1]. Type 2 diabetes mellitus (T2D) is characterized by insulin resistance (IR) and pancreatic beta cell dysfunction [2]. The third type of DM is called gestational DM (GDM), which develops during pregnancy (in the late second trimester or early in the third trimester) [3]. GDM is caused by any degree of glucose intolerance and defective pancreatic beta cells [3,4]. In addition, there are other types of DM such as maturity-onset diabetes of the young (MODY) and neonatal DM [4]. T2D is induced by the interaction of genetic and environmental or lifestyle risk factors [5]. Lifestyle or environmental risk factors include poor diet, reduced physical activity, depression, stress, and smoking [5]. In the last decade, genome-wide association studies (GWAs) have revealed the association of specific loci with diseases such as cancers, T2D, and cardiovascular disease [6,7,8,9,10,11]. For example, loci involved in the insulin signaling pathway may lead to IR and T2D [12,13]. The AKT protein is composed of 480 amino acid residues and comprises three domains: the pleckstrin homology (PH), middle catalytic (kinase) domain, and regulatory C-terminal disordered tail (C-tail) [14,15]. The PH domain anchors AKT to the membrane [15]. AKTs are classified into three isoforms (AKT1, AKT2, and AKT3) based on the differences in serine/threonine amino acid residues [15]. AKT1 is found ubiquitously, and AKT2 is found in insulin-sensitive tissues (liver, muscles, and adipose tissues) [15], whereas AKT3 is expressed in the testes and brain [15].

The serine/threonine kinase AKT2 is an effector protein in the insulin-signaling pathway and is connected to insulin metabolic actions [16]. It is encoded by the AKT2 gene (located on chromosome 19q13.1). The phosphoinositide 3-kinases (PI3Ks) are a family of lipid kinases that phosphorylate phosphatidylinositol, which is a component of eukaryotic cell membranes [17]. The AKT2 protein is expressed in various cell types within the human body, such as adipocytes, skeletal muscle cells, hepatocytes, and the pancreas [15,16]. The expression of AKT2 is stimulated by different stimuli such as cell stress, movement, hormones, and drugs that influence cellular metabolism [16]. There are two signaling pathways stimulated by insulin: the PI3K regulates metabolic function, and the mitogen-activated protein kinase (MAPK) signaling pathway is implicated in cell survival and mitogenesis [18].

PI3K/AKT signaling is crucial for regulating insulin responsiveness and other physiological and pathological processes, including growth, glucose homeostasis, lipid metabolism, protein synthesis, cell proliferation, survival, and angiogenesis [7,15,19,20,21]. In muscles, insulin regulates metabolism through the promotion of sugar uptake, glycogenesis, and the synthesis of protein via the PI3K/AKT signaling pathway [15]. It has been reported that about 90% of glucose metabolism is stimulated by insulin in muscles, which has important roles in energy homeostasis [15]. In adipose tissues, insulin–AKT signaling enhances the utilization of sugar and the synthesis of proteins and lipids [15,22]. In hepatocytes during the fed state, the PI3K/AKT signaling pathway decreases gluconeogenesis and glycogen breakdown and enhances glycogenesis and lipogenesis for peripheral utilization and storage [15,23].

IR and abnormalities in lipid metabolism have been associated with the dysfunction of AKT2 and other elements of the insulin signaling pathway [16,24]. Gene variations (p.G972R, and p.T608R) of insulin receptor substrate 1 (IRS-1), PI3K and (R274H, R208 K and R467W) and in AKT2 were reported to be associated with T2D, fasting hyperglycemia and postprandial hyperinsulinemia [24]. In addition, the p.Pro50Thr AKT2 mutation was associated with T2D risk in the Finnish population [25]. Moreover, gene polymorphisms in AKT1 and AKT2 were associated with T2D, polycystic ovarian syndrome (PCOS) and cancer in the Chinese population [26,27,28]. Furthermore, mutation (p.E17K) in AKT2 gene can result in defective insulin signaling pathway, which causes reduced levels of plasma insulin, ketone bodies, fatty acids and glucose [29]. It has been suggested that autonomous activation of the downstream insulin signaling pathway as a treatment strategy for patients carrying the mutation (p.E17K) should be considered [29], since the ATK2 protein has crucial functions in the insulin signaling pathway and defective insulin signaling pathway leads to metabolic syndrome, IR and T2D [30,31,32]. In the present study, we used bioinformatics tools to examine the non-synonymous SNP (nsSNPs) in the AKT2 protein that may influence its structure and function. 

## 2. Materials and Methods

**Figure 1:**
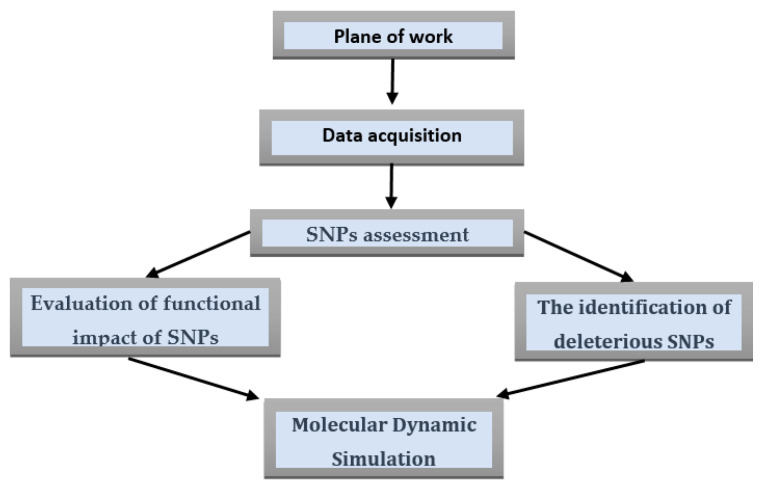
Workflow of the nsSNP detection and analysis. The nucleotide sequences are retrieved in FASTA format, and SNPs are assessed whether synonymous, nonsynonymous, or nonsense. Nonsynonymous SNPs (nsSNPs) are assessed on the subject of whether they have deleterious effects on the protein encoded by the gene by molecular dynamic simulation.

Figure 1 Schematic illustration of the steps of this study.

### 2.1. Dataset Download

The FASTA-formatted nucleotide sequence (Accession number: NG 012038.2) and amino acid sequence (NP 001229956.1) of the AKT2 protein were obtained from the National Center for Biotechnology Information (NCBI) database, which can be accessed at http://www.ncbi.nlm.nih.gov (accessed on 23 January 2023). The single nucleotide polymorphisms (SNPs) databases for the AKT2 (GLU17LYS) gene can be found in the NCBI’s SNP database (SNP), which is accessible at http://www.ncbi.nlm.nih.gov/snp/ (accessed on 23 January 2023). Similar data were acquired from the Online Mendelian Inheritance in Man (OMIM) database (http://www.omim.org (accessed on 23 January 2023)) regarding the AKT2 gene and protein.

### 2.2. Predicting the Effects of nsSNPs on AKT2 Protein Function

The assessment of potential effects of SNPs involved the utilization of several online tools and servers, namely the Sorting Intolerant From Tolerant (SIFT) Sequence, Polymorphism Phenotyping (poly-Phen2), Single Amino Acid Predication (SNAP2), and Protein Analysis Through Evolutionary Relationships (PANTHER). These tools and servers were employed to evaluate the impact of SNPs. Four servers were utilized to classify nsSNPs and assess their confidence ratings based on multiple inputs. The impact on protein function, specifically for non-synonymous single amino acid substitutions, can be determined by analyzing the predictions provided by SIFT, PolyPhen2, SNAP2, and PANTHER. These predictions classify the impact as either tolerable or damaging. The SIFT algorithm employs a tolerance index score to evaluate SNPs, whereby variations with scores below 0.05 are deemed deleterious. In contrast, the PolyPhen algorithm assigns a numerical score ranging from 0 to 1, where a score of 0 indicates a neutral effect and a score of 1 indicates the most severe negative effect. Another tool utilized in this study was SNAP2, which facilitates the comparison of genomes and provides predictions regarding functional consequences at the amino acid level. The SNAP2 algorithm utilizes a protein sequence and a set of Single Amino Acid Variants (SAVs) as input in order to forecast the impact of each substitution on the molecular function of the protein. The prediction scores span from a value of −100, indicating complete neutrality, to +100, indicating a significant effect. The final tool, known as PANTHER, employs a scoring system that spans from −1 to 1 in order to assess the projected influence of the input single nucleotide polymorphisms (SNPs) on protein functionality. The functionality of a single nucleotide polymorphism (SNP) can be deduced by evaluating its score. A positive score indicates that the SNP is functional and has an effect on protein function, while a negative score suggests that the SNP is non-functional. Greater absolute values are indicative of a more pronounced predicted impact on protein function. The single nucleotide polymorphisms (SNPs) of the AKT2 gene, which were found to be common among the fourth group of servers, were subsequently subjected to computational analysis.

### 2.3. The Prediction of Associations between nsSNPs and Disease

Predictor of human deleterious single nucleotide polymorphisms (PhD-SNP) (http://snps.biofold.org/phd-snp/phd-snp.html (accessed on 23 January 2023)) and SNPs&GO (http://snps-and-go.biocomp.unibo.it/snps-and-go/ (accessed on 23 January 2023)) were used for determining association of filtered SNPs with disease. The PhD-SNP online bioinformatics tool predicts the association between SNPs and diseases using machine learning methods. It assigns SNPs a score between 0 and 9 based on the probability of them resulting in a disease, classifying them as either disease-associated or neutral. The proportion of PhD accuracy SNP is 78% [33]. SNPs&GO is a precise tool that predicts disease-related amino acid changes at a particular position within a protein and offers functional classifications, with an overall prediction accuracy of 82% [34]. The UniProt accession number (P31751) of the AKT2 protein and the locations of the original and modified amino acids were the data needed by SNPs&GO. 

### 2.4. Predicting the Impact of SNPs on Protein Stability

Single nucleotide polymorphisms (SNPs) can modulate protein strength by either reducing or enhancing protein stability. In order to predict these effects with maximum confidence, a pair of tools was used. The tool known as I-Mutant, accessible at (http://folding.biofold.org/i-mutant/i-mutant2.0.html (accessed on 23 January 2023)), was utilized to forecast the effect of SNPs on the alteration of a protein’s stable state. It has been reported to possess an accuracy of up to 77% [35]. The I-Mutant algorithm was provided with the amino acid sequence of the AKT2 protein, along with information regarding the specific residues that had undergone mutations, including their respective positions. MUpro (https://mupro.proteomics.ics.uci.edu/ (accessed on 23 January 2023)) is a set of machine learning algorithms that detect alterations in protein states and stability due to amino acid mutations [36]. The inputs utilized by MUpro were identical to those employed by I-Mutant. However, MUpro additionally considers the locations of substitutions, along with the initial and altered amino acid residues.

### 2.5. Prediction of SNP Pathogencicty and Function Alteration on AKT2 Protein Using Mut-Pred Server

The Mut-pred web server (http://mutpred.mutdb.org/#qform (accessed on 23 January 2023)) is a valuable resource for forecasting the prospective influence of missense mutations on the functionality of proteins. The Mut-pred tool utilizes a comprehensive set of fifty distinct protein properties in order to determine the impact of substitutions. The objective is to forecast the probability of a particular missense mutation being either deleterious or benign. The requisite data for Mut-pred comprises the amino acid sequence of the protein that encompasses the mutation, in conjunction with the identity and position of the mutated residue. The resultant output furnishes an anticipated pathogenicity score, along with particulars regarding the precise characteristics of the protein function that are projected to be impacted by the mutation. A MutPred2 score exceeding 0.5 indicates the presence of a pathogenic mutation.

#### Analyzing Protein Sequence Conservation Using ConSurf Server

The examination of the conservation of the AKT2 protein was conducted utilizing the web-based programme ConSurf, accessible at http://ConSurf.tau.ac.il/ (accessed on 23 January 2023). This tool enables the high-throughput prediction of functional characteristics pertaining to specific protein regions. A conservation analysis value is assigned to each protein residue on a scale ranging from 1 to 9. Residues with scores of 1–3 indicate variabilities, scores of 4–6 indicate average conservation, and scores of 7–9 indicate highly conserved regions. In order to utilize the application, it is necessary to input the protein sequence in FASTA format.

### 2.6. Determination of the Active Binding Site Using PyMol Software

The software PyMol Version 2.0. was utilized to locate the binding site where alterations in amino acid composition took place. The utilization of pyMOL entails the examination of residues located in the active site. The aforementioned software is a tool that is open-source in nature and is utilized for the purpose of molecular visualization. It has the ability to produce three-dimensional images of small molecules with a superior level of quality. Thus, two potential mutants (A179T, L183Q) were selected from the 20 nsSNPs deemed to be the most deleterious mutants in order to conduct further analysis through molecular dynamics (MD) simulation. 

### 2.7. Performing Molecular Dynamics Simulations (MDS)

GROMACS version 2020.6 was used on a Google Collaboration pro notebook with high RAM and python 3.8 to run molecular dynamics (MD) calculations on both wildtype and mutant structures to look into the structural changes over time. For the first computation, the GROMACS-OPLS-AA force field was used. The systems were submerged in a cubic box filled with water molecules that had marginal radii of 1 nm (Å10), and the simulation box was neutralized by introducing 10 sodium Na+ ions using the GROMACS genion tool. The energy minimization was performed using the steepest descent algorithm with an energy step size of 0.01 and a maximum of 50000 iterations. To maintain a stable system, the Parrinello–Rahman (pcouple) pressure of 1 bar and a Berendsen temperature (tcouple) of 300 K were used. For temperature and pressure, the coupling constant was changed to 0.1 and 2.0 ps, respectively.

The electrostatic interactions in the system were computed using the Partial Mesh Ewald (PME) algorithm. The short-range cut-off for both Van der Waals (rvdw) and electrostatic (rcoulomb) interactions was set at 1.0 nm. The neighbor list (nstlist) was updated every 10 ps. All bond constraints, including heavy atom-H bonds, were maintained using the LINCS algorithm with a time step of 0.002 ps. The isothermal compressibility was set to 4.5 × 10^−5^. The system was equilibrated for 100 ps each in NPT (constant number of particles, pressure, and temperature) and NVT (constant number of particles, volume, and temperature) ensembles with the Berendsen temperature (tcouple) at 300 K and the Parrinello–Rahman (pcouple) pressure at 1 bar with coupling constants set to 0.1 ps and 2.0 ps for temperature and pressure, respectively. Subsequently, the native and mutant structures were subjected to 10 ns of molecular dynamics simulations, with trajectories recorded every 1 ps. The comparative analysis of structural deviations between the wildtype and mutant structures was conducted using various tools, such as g_rms, g_rmsf, g_sasa, g_Rg, and g_density, to analyze RMSD (root mean square deviation), RMSF (root mean square fluctuation), SASA (solvent accessible surface area), Rg (radius of gyration), pressure, temperature, and density plots. The application XMGRACE was used to create each plot.

## 3. Results

The present study examined the association of nsSNPs in the AKT2 gene and IR and T2D. Understanding the effects of on protein function is crucial for unraveling disease genetics and finding potential therapies. Computational methods utilize evolutionary conservation, protein structure, and function to predict nsSNP impacts on stability and structure. Investigation of the nsSNP AKT2’s effects offers insights into its role in IR and T2D development, aiding in understanding of the underlying disease mechanisms and identifying therapeutic targets. In addition, predicting nsSNP effects in the AKT2 gene can pinpoint high-risk individuals for tailored interventions and advance comprehension of genetic variation role in IR and T2D. Deciphering nsSNP impact on AKT2 could lead to personalized treatments targeting genetic variants, improving IR and glucose regulation for those at T2D risk. We present our results on AKT2 nsSNPs and their relevance to IR and T2D susceptibility, aiming to contribute to disease understanding and therapeutic development. 

### 3.1. The Anticipation of the Characteristics of SNPs

A summary of the results of the four different web servers, SIFT, PolyPhen2, SNAP2 and PANTHER, regarding the effects of different SNPs on the AKT2 protein are shown in Table 1.

The aforementioned table presents data pertaining to the SIFT score predictions for individual amino acid substitutions, which are utilized in the assessment of the effect on protein functionality. The data presented in the Table 1 suggest that the majority of substitutions are anticipated to be harmful, as evidenced by their SIFT score of less than 0.05, which implies a significant probability of impacting protein functionality. 

Nonetheless, certain substitutions are anticipated to be innocuous or slightly harmful, exhibiting elevated SIFT scores. In general, the SIFT score presents a valuable tool for forecasting the impact of SNPs on protein functionality. According to Table 1, the impact of the substitution is measured in terms of PolyPhen and SNAP2 scores, which are calculated by the respective programs. The PolyPhen score predicts the impact of the substitution as either “probably damaging”, “possibly damaging”, or “benign”. The SNAP2 score predicts the effect of the substitution as either “effect” or “no effect”.

To predict the SNPs’ nature with high confidence, SIFT, PolyPhen2, SNAP2, and PANTHER servers were employed concurrently. SIFT and PolyPhen2 received 533 missense nsSNPs in total. After consulting the databases it is integrated with, it offered each reported SNP an index score. A total of 142 (27%) of the nsSNPs were indicated by SIFT indexing to be harmful with a score of ≤0.05. Moreover, 99 out of 533 nsSNPs were reported to be highly harmful with a confidence level of 0.00. 

A total of 99 of the nsSNPs were classified as “probably damaging” by the PolyPhen indexing, which is based on structural data and multiple sequence alignment, and 43 as “possibly damaging”. As a result, 99 (18.6%) of nsSNPs had negative PolyPhen predictions. Combining data from SIFT and PolyPhen allowed the removal of 142 nsSNPs in order to produce the most accurate results. After that, SNAP2 and PANTHER servers received these 142 nsSNPs for additional verification. The 99 nsSNPs were then sent to the SNAP2 server to determine their effect. The findings showed that 75 nsSNPs were found to affect the protein functionally. A summary of the results related the nsSNP functional effects is shown in Table 1.

### 3.2. Identifying Disease-Linked SNPs

We employed SNP&GO and PhD-SNP tools to identify disease-associated SNPs and predict the connection between nsSNPs in AKT2 and IR and T2D, exploring their impact on AKT2 structure and function and IR and T2D development. The limited set of polymorphisms that have undergone filtration and reduction were subsequently evaluated to determine their association with diseases. Subsequently, 75 nsSNPs with a high level of confidence were submitted to SNP&GO and PhD-SNP. Both individuals computed a numerical value to classify every nsSNP as either benign or pathogenic. A single nsSNP was deemed neutral if its prediction score was less than 0.5, whereas a score greater than 0.5 indicated that the SNP was disease-causing. Out of a total of 75 nsSNPs, 47 variants were determined to have a neutral effect, while the remaining 28 nsSNPs were identified as having a causal association with disease. To reduce the number of nsSNPs, a cut off value of *p* ≥ 0.80 was utilized for PhD-SNP scores, resulting in a total of 20 nsSNPs. Table 2 depicts the outcomes that were subjected to filtration.

### 3.3. The Prognostication of the Impact of Protein Stability

We used the I-Mutant and MuPro servers to try to figure out what would happen to the structure of 20 possible nsSNPs. Based on the results, 20 nsSNPs were thought to stop proteins from working by making them less stable. Moreover, as per the Mut-pro server, it was indicated that only 3 nsSNP out of 20 showed an increase on the protein’s functionality due to structure impact. The findings are presented in Table 3.

### 3.4. Prediction of the Pathogenicity and Underlying Functional Alterations of nsSNP Effects on AKT2 Protein

We utilized The Mut-pred web server to predict the pathogenicity and underlying functional changes caused by nsSNPs on the AKT2 protein. This approach aids in understanding the potential impact of these genetic variations on protein function and their association with disease, such as T2D related to insulin resistance. The Mut-pred web server offers valuable insights into the molecular mechanisms linking nsSNPs in AKT2 to disease susceptibility and provides a foundation for further investigation. The Mut-pred web server generates outcomes that aid in predicting the probable influence of missense mutations on protein function that is linked with diseases. Furthermore, Mut-pred provides details regarding the particular characteristics of the protein that are anticipated to be impacted by the mutation, encompassing solvent accessibility, secondary structure, and sequence conservation. According to the results presented in Table 4 of Mut-pred, all 20 nsSNP were found to have an impact on protein function. However, it was observed that 10 out of the 20 nsSNPs had a solely negative effect on the AKT2 protein, attributable to alterations in the protein’s structure and characteristics.

### 3.5. Study of Sequence Conservation

**Figure 2:**
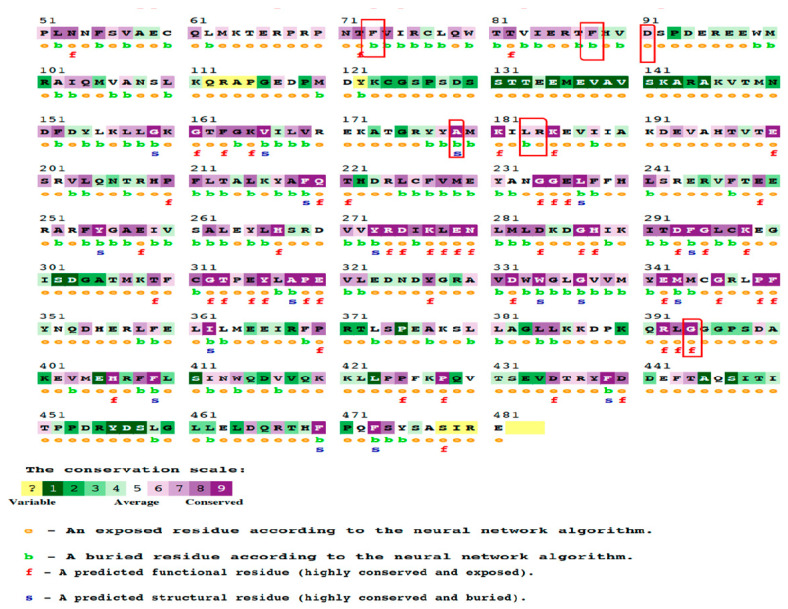
The ConSurf analysis yielded results pertaining to the conservation of residues. The ConSurf results display a range of colours that correspond to the level of confidence regarding sequence conservation. In this colour scheme, the sky-blue colour represents variable residues, while the dark purple colour represents highly conserved residues.

The ConSurf server was utilized to investigate the preservation of 20 nsSNPs that are associated with T2D. As per the findings, a set of three nsSNPs exhibited variability, whereas four nsSNPs demonstrated a modest conservation level. A set of nsSNPs that were determined to be conserved was analyzed, revealing that seven of them displayed both structural and spatial conservation, and they were located in buried regions. Six nsSNPs were predicted to be functionally conserved and exposed. The ConSurf tool employs chromatic schemes to represent the extent of sequence conservation and offers predictions about the nature and location of modifications on a numerical scale ranging from 1 to 9. Table 5 and Figure 2 provide a comprehensive summary of the 20 nsSNPs that were detected, including their functional and structural implications, as well as their phylogenetic conservation scores. 

Figure 2: Analyses of the AKT2 structure showing alanine (A) 179 and leucine (L) 183 are conserved amino residues.

### 3.6. Molecular Dynamics Simulation

Through these simulations, we gained valuable insights into the dynamic behavior of the protein, its interactions, and conformational changes that may influence AKT2 structure and function that may lead IR and T2D.

#### 3.6.1. Temperature, Pressure and Density

**Figure 3:**
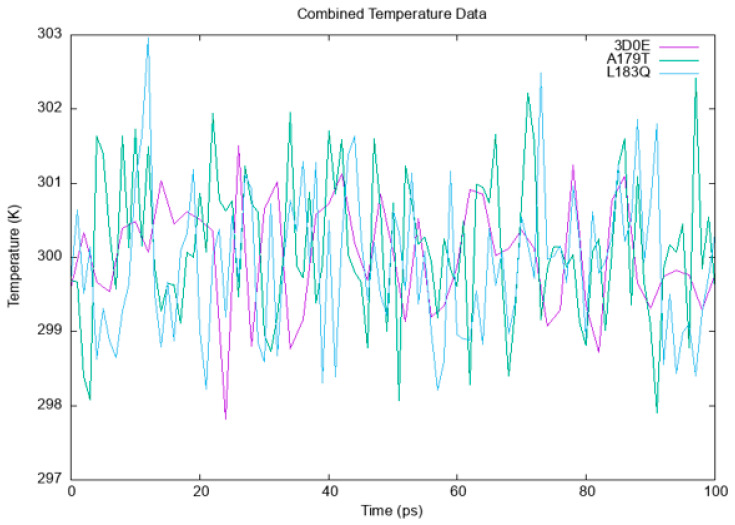
The variations in temperature over time for both the wildtype and mutant forms of AKT2.

**Figure 4:**
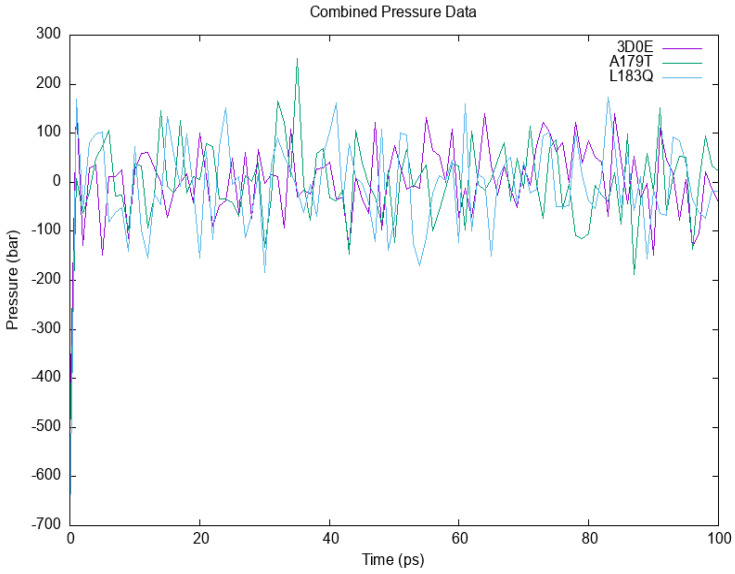
The variations in pressure over time for both the wildtype and mutant forms of AKT2.

**Figure 5:**
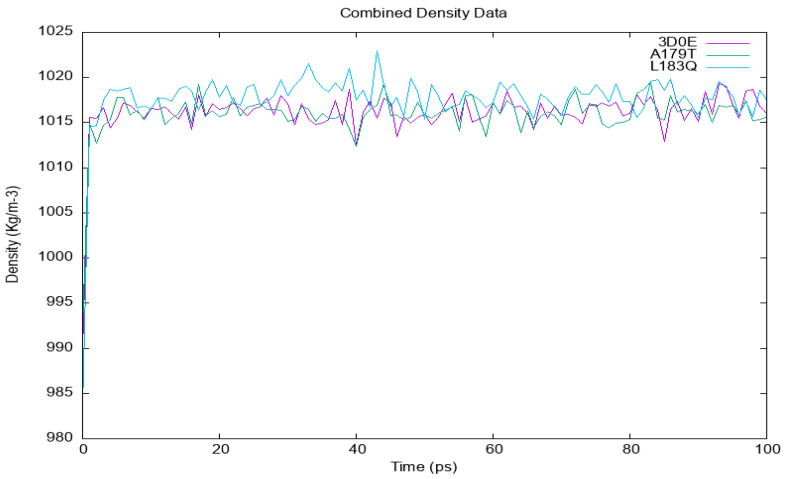
The variations in density over time for the wildtype and mutant AKT2 protein.

Solvents and ions were then balanced around the protein structures after energy minimization. NVT (thermostat) and NPT (bariostat) equilibrations were carried out to make sure that the molecules were oriented correctly within the structures. In an isothermal–isobaric ensemble, these two phases were used to stabilize the systems’ temperature and pressure, respectively. The computational tool known as “gms/grompp” was utilized to perform calculations for the NVT and NPT ensembles of the system. The equilibrated process involves temperature, pressure, and density, leading to the establishment of a density plot from NPT. Figure 3 depicts the temperature profiles of the protein systems in their native and mutant forms at a temperature of 300 Kelvin. The plot illustrates significant temperature fluctuations in the conformations during the 100 ps equilibrium phase. Nonetheless, this conduct was not entirely unforeseen, given that the mean temperature of the majority of the conformations fell within a favorable range, with the native structure exhibiting an average temperature of 301 K, and the mutant A179T and L183Q displaying temperatures of 301.5 and 302.9 K, respectively (Figure 3). Likewise, a pressure–time graph (Figure 4) and a density–time graph (illustrated in Figure 5) were generated. 

Figure 3: A graph illustrating the variations in temperature over time for both the wildtype and mutant forms of AKT2, as simulated using the GROMACS software. https://www.gromacs.org/. The wildtype is visually represented by the colour purple, while the A179T mutant is depicted in green, and the L183Q mutant is represented by the colour blue.

Figure 4 contains a graph illustrating the variations in pressure over time for both the wildtype and mutant forms of AKT2, as simulated using the GROMACS software. The wildtype is visually represented by the colour purple, while the A179T mutant is depicted in green, and the L183Q mutant is represented by the color blue.

The pressure–time graph (Figure 4) indicated that the wildtype structure exhibited peaks above 110 bars, while the A179T and L183Q mutant structures displayed higher peaks of 260 bars and 180 bars, respectively. The plot for mutant A179T exhibits a local minimum of −500 bar, which deviates from the native structure’s value of −350 bars and that of L183Q is −650 bars. The graph depicting pressure over time indicated that the mean pressure value for all conformations in the native structure was 95 bars. However, for A179T and L183Q, the average pressure values were 105 bars and 100 bar, respectively. The present study examines the density values of the native akt2 protein and its mutant variant A179T over a period of 100 ps (Figure 5). 

Figure 5 contains a graph illustrating the variations in density over time for both the wildtype and mutant forms of AKT2, as simulated using the GROMACS software. The wildtype is visually represented by the purple color, while the A179T mutant is depicted in green, and the L183Q mutant is represented by the blue color.

The average density value observed for both the native protein and the mutant variant was 1015 kg/m^−3^, with minor fluctuations. However, it is noteworthy that the mutant A179T exhibited a lower initial density value of 985 kg/m^−3^. The L183Q mutant displays analogous fluctuations in pattern to the native AKT2 and mutant A179T; however, it maintains a higher value of 1020.5 Kg/m^−3^.

#### 3.6.2. Root-Mean-Square Deviation (RMSD) and Root-Mean-Square-Fluctuation (RMSF)

**Figure 6:**
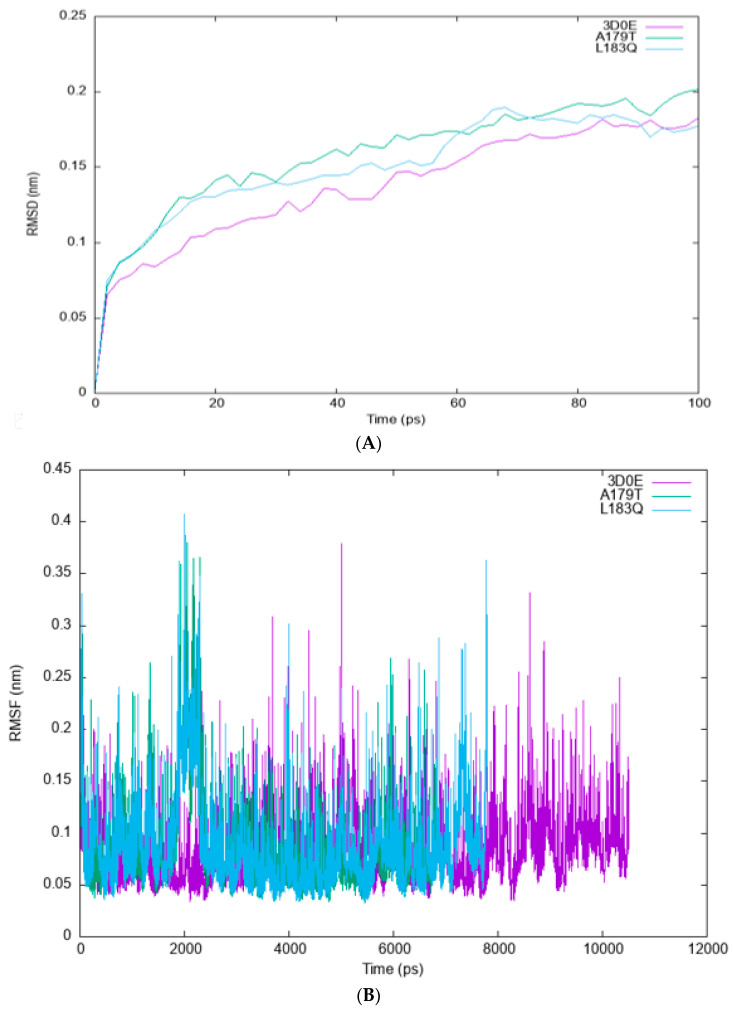
The root-mean-square deviation (RMSD) (**A**) and root-mean-square fluctuation (RMSF) (**B**) graphs were generated for the backbone atoms of both the wildtype and mutant variants of the AKT2 protein. These simulations were conducted using GROMACS version 5.1.2. The wildtype is visually represented by the colour purple, while the A179T mutant is depicted in green and the L183Q mutant is depicted in blue.

Comparative analysis of the RMSF and RMSD of AKT2 wildtype and its A179T and L183Q mutations was performed. To compare and depict the structural and functional behavior of the wildtype AKT2 gene and its two mutant forms, A179T and L183Q, the current work used a molecular dynamics simulation method. The RMSD was used to evaluate the trajectories of the native and mutant forms of the AKT2 protein. The trajectory file for the C-alpha backbone least-squares fit model and g_rms were used to construct the RMSD graphs shown in Figure 6A.

Figure 6 shows the root-mean-square deviation (RMSD) and root-mean-square fluctuation (RMSF) for the wildtype and the mutants of the AKT2 protein. 

The initial conformations of the three structures exhibited close proximity; however, a sudden deviation was observed at 10 ps, resulting in an RMSD value of 0.11 nm for the two mutants.

The wildtype structure achieved its highest RMSD value at 8 ps, while the A179T and L183Q mutants reached their maximum values at 14 ps and 15 ps, respectively. The RMSD graph comparing the wildtype AKT2 gene to its A179T and L183Q mutants, which were aligned to the backbone, indicated that the wildtype AKT2 gene exhibited greater stability than the A179T and L183Q mutants. Subsequently, the aforementioned mutants have been observed to impact the protein dynamics of the wildtype AKT2. This is evidenced by alterations in the RMSD graph of the backbone of the mutants, thereby providing a suitable basis for subsequent analysis. The analysis of RMSF values of native and mutant residues was conducted to ascertain the impact of mutations on the dynamic behavior at the atomic level (Figure 6B). The RMSF of the native residues was found to range from 0.041 nm to 0.38 nm, with an average value of 0.21 nm. The RMSF values for the A179T and L183Q residues were determined to range from 0.032 to 0.38 and 0.04 to 0.41, respectively, with average values of 0.22 and 0.23.

#### 3.6.3. Radius of Gyration (Rg) and Solvent Accessibility Surface Area (SASA)

**Figure 7:**
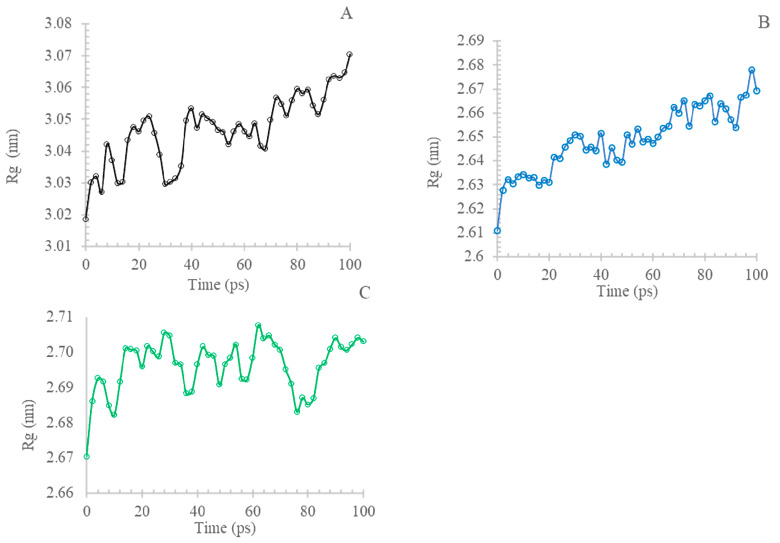
The radius of gyration (Rg) of Cα atoms of native and mutant-type AKT2 protein. Wildtype (**A**) is shown in black, A179T (**B**) mutant is shown in blue and L183Q (**C**) is shown in green.

**Figure 8:**
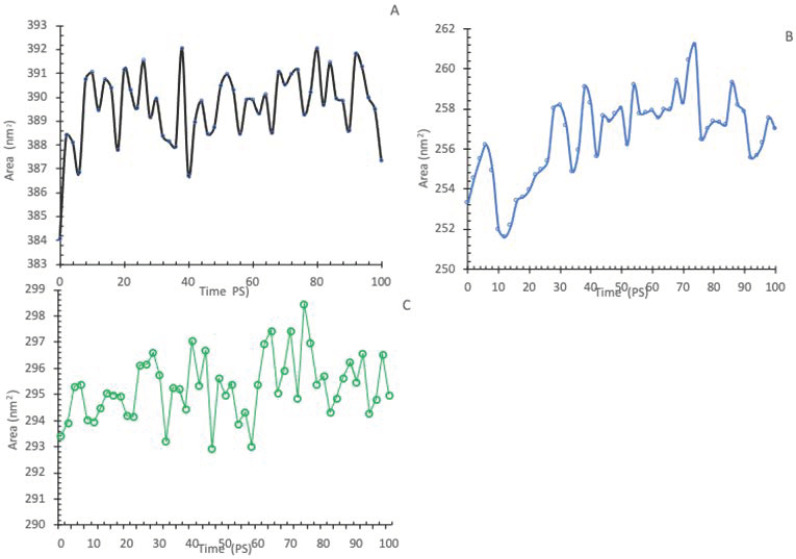
Solvent accessible surface area of wildtype (**A**) and mutant-type AKT protein. Wildtype is shown in black, A179T (**B**) mutant is shown in Blue and L183Q (**C**) is shown in green.

Analyzing the Radius of Gyration (Rg) and Solvent Accessibility Surface Area (SASA) of both the wildtype and the A179T and L183Q mutations in AKT2 is vital in understanding their impact on AKT2 structure and function. These structural metrics offer insights into the compactness and exposure of amino acids, shedding light on potential conformational changes that contribute to the complex relationship between these mutations and IR and T2D. The concepts of radius of gyration and SASA are of interest in various fields of study. The concept of Rg pertains to the root-mean-square distance that all atoms occupy from their shared center, as determined by their mass and weight. This makes it easier to understand all of the components of protein analysis. Figure 7 compares the normal and mutant protein structures at a temperature of 300 K by plotting the radius of gyration for alpha carbon atoms over time. Upon completion of the simulation, it was observed that the structure of the two mutants, A179T (Figure 7B) and L183Q (Figure 7C), exhibited normal behavior like that of the wildtype (Figure 7A), characterized by consistently low Rg values (<2.63 nm and 2.68, respectively). In contrast, the Rg values for the native structures remaining higher ranged from 3.09 nm to 3.05 nm. SASA refers to the degree of accessibility of the biomolecular surface area to solvent molecules. A reduction in the SASA value signifies a contraction in the structure and a decrease in the surface area. Figure 8 illustrates that the native structure exhibited a significantly elevated SASA value, which ranged from 305 nm^2^ to 317.5 nm^2^, with an average of 315 nm^2^. The SASA values for A179T (Figure 8B) and L183Q (Figure 8B) were observed within the range of 253 nm^2^ to 260 nm^2^, and 293.5 nm^2^ to 298.5 nm^2^, with an average of 256 nm^2^ and 296 nm^2^, respectively.

Figure 7 shows the radius of gyration (Rg) of Cα atoms of native and mutant-type AKT2 protein. 

Figure 8: Solvent accessible surface area of wildtype and mutant AKT2 protein.

**Figure 9:**
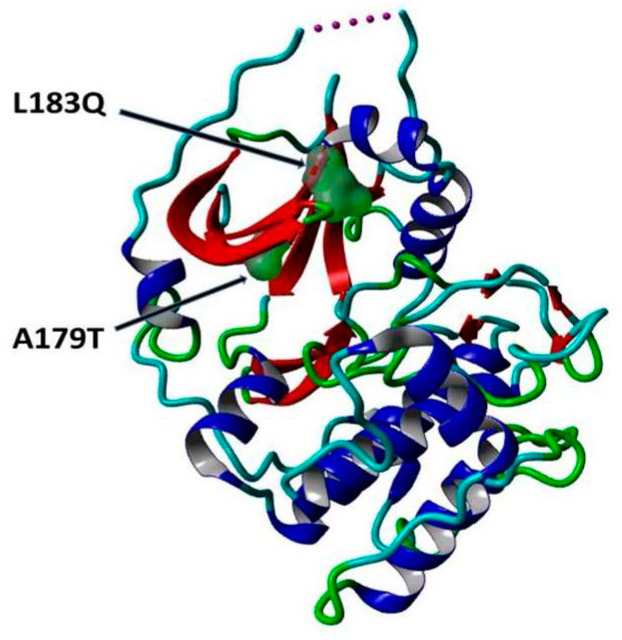
The cartoon structure of the AKT2 protein (PDB ID: 3D0E) showing the sites of the mutations p.A179T and p.L183Q with surface presentations indicated with arrows. This figure is prepared using YASARA view.

Figure 9 shows the three-dimensional structure of AKT2 with the sites of mutations indicated with arrows. 

## 4. Discussion

The prevalence of DM is increasing worldwide and it has a serious socioeconomic burden [37]. T2D represents about 90% of all cases of DM [38]. T2D is induced by insulin resistance (IR) or impaired insulin action and pancreatic beta cell demise [39]. IR is a reduced cellular metabolic responsiveness to insulin and a defective lowering effect of circulating or injected insulin on blood sugar [40]. Impaired insulin signaling, for example, because of a defect of AKT2 or a dysfunction of PI3K/AKT signal transduction, can lead to IR [41]. IR is associated with the risk factors of the metabolic syndrome [42]. These risk factor include obesity and dyslipidemia, hyperinsulinemia, high blood pressure, and elevated blood sugar [42,43]. IR and metabolic syndrome are associated with increased susceptibility to T2D and cerebrocardiovascular disease [42,43].

The serine/threonine kinase AKT2, also called protein kinase B (PKB), is implicated in the regulation of insulin signaling pathway. Insulin has important roles in the metabolism of glucose, proteins, and fats [44]. AKT2 is an important mediator in the PI3K pathway, and it binds different downstream proteins that influence cellular metabolism and other process [18]. Gene variations in insulin signaling genes or genes involved in carbohydrate metabolism were reported to be associated with T2D [7,8,45,46]. Insulin enhances the glucose uptake from the circulation in muscles and fat tissues via enhancing the movement of glucose transporter (GLUT4) from their intracellular compartment to the plasma membrane [47]. This movement of GLUT4 from their pool and infusion in the plasma membrane is mediated by AKT2 and its downstream effectors [48]. In the liver, insulin signaling mediated by the AKT2 protein is very important for the inhibition of hepatic glucose synthesis and stimulating lipogenesis [16,49]. Defective AKT2 protein in the pancreatic beta cells causes decreased insulin secretion, which has negative effects on insulin levels in the peripheral tissues [16]. Moreover, it induces insulin insensitively in muscles, adipocytes, and hepatocytes, causing decreased glycogen absorption in muscle cells, reduces glycogenesis in hepatocytes, enhances the breakdown of lipids and induces IR [16]. IR, or the impaired insulin action, that is, irresponsiveness to the insulin in adipose tissues, muscles, liver and other tissues, represents an important component of the pathophysiology of metabolic syndrome [50,51]. Previous studies reported that impaired insulin action is a step preceding the induction of T2D and comorbidities such as abdominal obesity, hyperlipidemia, hepatic steatosis, PCOS, increased blood pressure, and cardiovascular disease [16,50,52]. AKT2 gene variations were associated with PCOS [27], T2D [25], and cancer [53]. The AKT2 variant with alternative splicing is proposed to code the AKT2 protein with a defective C-terminal regulatory region without the hydrophobic motif regulatory site [54]. In the present study, a computational investigation was conducted to assess the effect of nsSNPs on the AKT2 protein structure and function that may lead to the development of IR and T2D. 

Our study identified 20 variants as the most detrimental effect based on the collective outcomes of various analytical tools. Then, two potential mutants (A179T, L183Q) were selected from the 20 nsSNPs deemed to be most pathogenic mutants for further investigation through molecular dynamics (MD) simulation. The identified mutations were observed to modify the protein’s stability and function properties, which may have an impact on its biological activity. A molecular dynamics simulation was performed to evaluate the disparities in protein dynamics between the wildtype and mutant AKT2 proteins. The simulation was conducted over a period of 100 ps, during which a range of parameters, including temperature, pressure, density, RMSD, RMSF, SASA, and Rg, were evaluated and scrutinized. Comparisons were performed to investigate the functional characteristics and stability of the protein structures. Upon conducting minimization, marginal variances in temperature, pressure, and density were observed between the native and mutant conformations over the course of the simulation, although the mutant p.L183Q revealed slight increases in these parameters. The RMSD plots of backbone atoms indicated that the wildtype structure demonstrated a higher degree of stability in comparison to the mutants. The RMSF plots indicated that the native and mutant structures exhibited the greatest level of flexibility. The RMSF calculations, which were averaged across the residual elements and trajectories, indicated that the fluctuation of atoms within the structures was notably greater in the first third. The investigation encompassed an analysis of the radius of gyration and SASA to ascertain the impact of the mutant protein structure’s unfolding on the solubility of surface area and the activity of the protein. A higher Rg value is indicative of reduced stability. The present findings suggest that the native structure exhibited a narrower range of Rg values in comparison to the mutants. Moreover, the solvent accessible surface area of the wildtype AKT2 is different from the mutants of the AKT2 protein. This may indicate that the two mutations (p.A179T and p.L183Q) lead to a reduction in AKT2 stability and functionality. 

Alanine (A) is the second simplest amino acid; it is hydrophobic. Meanwhile, threonine contains a hydroxyl group; it is a hydrophilic [55]. In p.A179T, the substitution of alanine by threonine can have a profound effect on the AKT2 protein structure and function [56]. Moreover, p.A179T may lead to a loss of helix according to the Mut-pred web server result, which may affect the AKT2 protein. In addition, in p.L183Q, the substitution of leucine (L), a hydrophobic amino acid, by glutamine (Q), a hydrophilic amino acid [55], may exert an impact on the AKT2 protein structure and function. Furthermore, according to the Mut-pred web server, p.L183Q may lead to the loss of acetylation at lysine (K) 181 residue, which may influence the AKT2 protein. This result may be consistent with that of a study which reported that a missense mutation (p.R274H) in the AKT2 generates dysfunctional AKT2 and results in impaired insulin action, hyperinsulinemia, and T2D [29]. Our results are also in agreement with those of a study that reported that a partial loss of function in p.Pro50Thr mutation in AKT2 causes reduced glucose uptake in insulin-sensitive tissues, and it is associated with elevated fasting blood insulin and risk of T2D [31]. To the best of our knowledge, this is the first study highlighting the importance of these mutations (p.A179T and p.L183Q) on the AKT2 protein structure and function. These results need further verification before introduction in the clinic setting via well-designed large-scale case–control, protein–protein interaction (PPI) and protein functional studies [57,58,59,60] to examine the effect of these mutations on the AKT2 protein structure, function and the insulin signaling pathway.

## 5. Conclusions

In this study, we examined the potential association of non-synonymous SNP (nsSNPs) in the AKT2 gene with IR and T2D. Results indicated that two mutations, p.A179T and p.L183Q, may cause a reduction in AKT2 protein stability and functionality which can be a cause for IR, metabolic syndrome and T2D. These results require further verification in protein functional, PPI and large case–control studies. After being verified, these results can be used in genetic testing for T2D in risk stratification for treatment or prevention strategies. 

## Figures and Tables

**Table 1:** The effects of amino acid substitution on the AKT2 gene as predicted by SIFT (deleterious or tolerated), PolyPhen (damaging or benign), SNAP2 and PANTHER.

				SIFT		Polyphen		SNAP2		PANTHER	
Variant ID	Allele	Protein	Mutation	Score	Prediction	Score	Prediction	Effects	Expected Accuracy %	pdel	Prediction
rs1017033490	C|T	ENST00000311278.10	R480H	0.00	deleterious	0.988	probably damaging	Effect	65%	0.57	probably damaging
rs769806554	G|A	ENST00000311278.10	R480C	0.00	deleterious	0.992	probably damaging	Effect	63%	0.57	probably damaging
rs769806554	G|C	ENST00000311278.10	R480G	0.00	deleterious	0.986	probably damaging	Effect	75%	0.57	probably damaging
rs1447335111	T|C	ENST00000311278.10	Y475C	0.00	deleterious	1	probably damaging	Effect	80%	0.85	probably damaging
rs1447335111	T|G	ENST00000311278.10	Y475S	0.00	deleterious	0.991	probably damaging	Effect	91%	0.85	probably damaging
rs1368133745	A|T	ENST00000311278.10	F470I	0.00	deleterious	1	probably damaging	Effect	80%	0.85	probably damaging
rs1251045508	G|C	ENST00000311278.10	S432C	0.01	deleterious	0.999	probably damaging	Effect	61%	0.85	probably damaging
rs757239082	A|G	ENST00000311278.10	M404T	0.09	deleterious	0.644	possible damaging	Effect	82%	0.85	probably damaging
rs757239082	A|C	ENST00000311278.10	V403G	0.00	deleterious	1	probably damaging	Effect	80%	0.57	probably damaging
rs1278736092	C|G	ENST00000311278.10	G396R	0.01	deleterious	0.999	probably damaging	Effect	53%	0.86	probably damaging
rs1207581157	C|T	ENST00000311278.10	G396R	0.01	deleterious	0.999	probably damaging	Effect	53%	0.86	probably damaging
rs1316408057	C|A	ENST00000311278.10	G395W	0.01	deleterious	1	probably damaging	Effect	66%	0.74	probably damaging
rs1371711287	C|A	ENST00000311278.10	G394C	0.00	deleterious	1	probably damaging	Effect	63%	0.89	probably damaging
rs867381867	A|T	ENST00000311278.10	L385H	0.00	deleterious	1	probably damaging	Effect	91%	0.89	probably damaging
rs767039609	G|A	ENST00000311278.10	A377V	0.00	deleterious	1	probably damaging	Effect	63%	0.86	probably damaging
rs752215436	G|A	ENST00000311278.10	P370L	0.00	deleterious	1	probably damaging	Effect	53%	0.89	probably damaging
rs754942500	G|A	ENST00000311278.10	R357C	0.00	deleterious	1	possible damaging	Effect	72%	0.57	probably damaging
rs749701848	G|A	ENST00000311278.10	P349L	0.00	deleterious	0.998	probably damaging	Effect	53%	0.89	probably damaging
rs1470884234	G|A	ENST00000311278.10	R347C	0.01	deleterious	0.985	probably damaging	Effect	85%	0.85	probably damaging
rs1198321649	C|T	ENST00000311278.10	G346S	0.01	deleterious	1	probably damaging	Effect	66%	0.89	probably damaging
rs1485158704	A|T	ENST00000311278.10	M344K	0.01	deleterious	1	probably damaging	Effect	71%	0.85	probably damaging
rs765026585	G|A	ENST00000311278.10	R329W	0.00	deleterious	1	possible damaging	Effect	78%	0.86	probably damaging
rs765026585	C|A	ENST00000311278.10	D326Y	0.03	deleterious	1	probably damaging	Effect	80%	0.85	probably damaging
rs1257580103	G|A	ENST00000311278.10	A318V	0.00	deleterious	1	probably damaging	Effect	71%	0.89	probably damaging
rs1458251691	T|C	ENST00000311278.10	E315G	0.00	deleterious	0.995	probably damaging	Effect	75%	0.86	probably damaging
rs1437512007	G|A	ENST00000311278.10	P314L	0.00	deleterious	0.998	probably damaging	Effect	80%	0.86	probably damaging
rs753989242	G|C	ENST00000311278.10	P314A	0.00	deleterious	1	probably damaging	Effect	80%	0.86	probably damaging
rs529742537	T|C	ENST00000311278.10	M307V	0.02	deleterious	0.998	probably damaging	Effect	71%	0.74	probably damaging
rs200395003	C|T	ENST00000311278.10	D284N	0.04	deleterious	0.994	possible damaging	Effect	53%	0.86	probably damaging
rs777146223	C|T	ENST00000311278.10	R274H	0.00	deleterious	0.994	probably damaging	Effect	85%	0.89	probably damaging
rs139506765	G|A	ENST00000311278.10	R274C	0.00	deleterious	0.999	probably damaging	Effect	95%	0.89	probably damaging
rs778561687	C|T	ENST00000311278.10	V271M	0.00	deleterious	1	probably damaging	Effect	95%	0.74	probably damaging
rs778561687	G|A	ENST00000311278.10	R269W	0.02	deleterious	0.948	probably damaging	Effect	80%	0.57	probably damaging
rs745550650	A|T	ENST00000311278.10	Y265N	0.01	deleterious	0.985	probably damaging	Effect	85%	0.86	probably damaging
rs1568518681	G|C	ENST00000311278.10	A252G	0.01	deleterious	1	probably damaging	Effect	85%	0.78	probably damaging
rs930976777	G|A	ENST00000311278.10	R251W	0.00	deleterious	0.9	probably damaging	Effect	66%	0.86	probably damaging
rs1568518786	G|A	ENST00000311278.10	R245C	0.01	deleterious	1	probably damaging	Effect	75%	0.89	probably damaging
rs753149827	G|A	ENST00000311278.10	R243W	0.00	deleterious	0.976	probably damaging	Effect	82%	0.85	probably damaging
rs756750912	T|C	ENST00000311278.10	N233D	0.01	deleterious	0.992	probably damaging	Effect	66%	0.86	probably damaging
rs1020231672	A|G	ENST00000311278.10	Y231H	0.00	deleterious	0.999	probably damaging	Effect	63%	0.86	probably damaging
rs145305228	T|G	ENST00000311278.10	T221P	0.00	deleterious	1	probably damaging	Effect	85%	0.89	probably damaging
rs1169786300	G|C	ENST00000311278.10	L215V	0.00	deleterious	0.997	probably damaging	Effect	80%	0.86	probably damaging
rs372625056	A|G	ENST00000311278.10	L212P	0.00	deleterious	0.997	probably damaging	Effect	71%	0.86	probably damaging
rs1166756826	G|C	ENST00000311278.10	P210R	0.01	deleterious	1	probably damaging	Effect	95%	0.89	probably damaging
rs1422114933	G|A	ENST00000311278.10	R202W	0.00	deleterious	1	probably damaging	Effect	71%	0.85	probably damaging
rs1004075798	T|C	ENST00000311278.10	T197A	0.01	deleterious	0.875	probably damaging	Effect	85%	0.86	probably damaging
rs776561628	G|A	ENST00000311278.10	R184W	0.00	deleterious	0.76	possible damaging	Effect	71%	0.57	probably damaging
rs1568519097	A|T	ENST00000311278.10	L183Q	0.00	deleterious	0.996	probably damaging	Effect	71%	0.86	probably damaging
rs1323113005	C|T	ENST00000311278.10	A179T	0.01	deleterious	0.995	probably damaging	Effect	66%	0.89	probably damaging
rs763266151	T|G	ENST00000311278.10	T174P	0.01	deleterious	0.99	probably damaging	Effect	91%	0.85	probably damaging
rs144395843	G|A	ENST00000311278.10	R170W	0.00	deleterious	0.994	probably damaging	Effect	85%	0.57	probably damaging
rs980537301	C|T	ENST00000311278.10	V169M	0.01	deleterious	0.997	probably damaging	Effect	80%	0.85	probably damaging
rs140987550	A|G	ENST00000311278.10	M149T	0.02	deleterious	0.907	probably damaging	Effect	71%	0.74	probably damaging
rs762379584	G|A	ENST00000311278.10	S126F	0.00	deleterious	0.99	probably damaging	Effect	66%	0.74	probably damaging
rs121434593	A|G	ENST00000311278.10	I103T	0.02	deleterious	0.823	probably damaging	Effect	59%	0.85	probably damaging
rs774959037	T|A	ENST00000311278.10	I103F	0.02	deleterious	0.999	probably damaging	Effect	66%	0.85	probably damaging
rs1304063423	C|A	ENST00000311278.10	R101W	0.02	deleterious	0.913	probably damaging	Effect	59%	0.5	possibly damaging
rs1347953319	C|T	ENST00000311278.10	E97G	0.02	deleterious	0.99	probably damaging	Effect	71%	0.85	probably damaging
rs757245351	C/A	ENST00000311278.10	D91Y	0.00	deleterious	0.732	possible damaging	Effect	66%	0.74	probably damaging
rs758367800	A/G	ENST00000311278.10	F88L	0.01	deleterious	0.999	probably damaging	Effect	66%	0.86	probably damaging
rs747207035	C/T	ENST00000311278.10	E85K	0.02	deleterious	0.945	probably damaging	Effect	66%	0.86	probably damaging
rs1207387979	C/A/T	ENST00000311278.10	V74F	0.04	deleterious	1	probably damaging	Effect	59%	0.5	possibly damaging
rs145907048	A/C	ENST00000311278.10	F73V	0.00	deleterious	0.93	probably damaging	Effect	75%	0.85	probably damaging
rs1428621686	G/A	ENST00000311278.10	P68L	0.01	deleterious	1	probably damaging	Effect	53%	0.85	probably damaging
rs773553776	G/A/C	ENST00000311278.10	P51R	0.03	deleterious	0.825	probably damaging	Effect	75%	0.85	probably damaging
rs771461640	G/C	ENST00000311278.10	P51A	0.03	deleterious	0.999	probably damaging	Effect	63%	0.85	probably damaging
rs1367814764	T/G	ENST00000311278.10	Y26S	0.00	deleterious	0.791	possible damaging	Effect	91%	0.85	probably damaging
rs775105991	C/T	ENST00000311278.10	R25Q	0.00	deleterious	1	probably damaging	Effect	91%	0.86	probably damaging
rs1363221582	A/G	ENST00000311278.10	I19T	0.00	deleterious	0.987	probably damaging	Effect	80%	0.86	probably damaging
rs763896696	C/A/T	ENST00000311278.10	E17K	0.05	deleterious	1	probably damaging	Effect	85%	0.86	probably damaging
rs988297708	G/A	ENST00000311278.10	R15C	0.00	deleterious	1	probably damaging	Effect	80%	0.86	probably damaging
rs1227145174	T/G	ENST00000311278.10	K14T	0.00	deleterious	1	probably damaging	Effect	91%	0.86	probably damaging
rs754286414	C/T	ENST00000311278.10	E9K	0.00	deleterious	0.998	probably damaging	Effect	91%	0.85	probably damaging
rs1373751808	T/A/C	ENST00000311278.10	I7F	0.02	deleterious	0.93	probably damaging	Effect	71%	0.27	probably benign
rs746407532	C/A	ENST00000311278.10	V6F	0.01	deleterious	0.752	possible damaging	Effect	66%	0.57	probably damaging

**Table 2:** Disease association of the non-synonymous SNP (nsSNPs) in AKT2 protein as predicted by SNP&GO and PhD-SN.

			PhD-SNP		SNP&GO	
Variant ID	Allele	Mutation	Score	Prediction	Score	Prediction
rs1447335111	T|C	Y475C	3	Disease	1	Disease
rs1447335111	T|G	Y475S	6	Disease	2	Disease
rs1368133745	A|T	F470I	8	Disease	5	Disease
rs1371711287	C|A	G394C	4	Disease	4	Disease
rs749701848	G/A	P349L	4	Disease	6	Disease
rs1198321649	C/T	G346S	0	Disease	7	Disease
rs1485158704	A/T	M344K	9	Disease	8	Disease
rs765026585	C/A	D326Y	4	Disease	5	Disease
rs1257580103	G/A	A318V	7	Disease	6	Disease
rs930976777	G/A	R251W	2	Disease	5	Disease
rs753149827	G/A	R243W	2	Disease	2	Disease
rs145305228	T/G	T221P	5	Disease	3	Disease
rs1166756826	G/C	P210R	4	Disease	6	Disease
rs1422114933	G/A	R202W	1	Disease	3	Disease
rs1568519097	A/T	L183Q	0	Disease	4	Disease
rs1323113005	C/T	A179T	2	Disease	7	Disease
rs763266151	T/G	T174P	7	Disease	4	Disease
rs758367800	A/G	F88L	8	Disease	1	Disease
rs145907048	A/C	F73V	2	Disease	4	Disease
rs775105991	C/T	R25Q	7	Disease	3	Disease

**Table 3:** Predication of the effects of non-synonymous SNP (nsSNPs) in the AKT2 protein stability (increasing or decreasing).

Variant ID	Allele	Mutation	I-Mutant	MUpro
rs1447335111	T|C	Y475C	Decrease	Decrease
rs1447335111	T|G	Y475S	Decrease	Decrease
rs1368133745	A|T	F470I	Decrease	Decrease
rs1371711287	C|A	G394C	Decrease	Decrease
rs749701848	G/A	P349L	Decrease	Decrease
rs1198321649	C|T	G346S	Decrease	Decrease
rs1485158704	A|T	M344K	Decrease	Decrease
rs765026585	C|A	D326Y	Decrease	Decrease
rs1257580103	G|A	A318V	Decrease	Increase
rs930976777	G|A	R251W	Decrease	Decrease
rs753149827	G|A	R243W	Decrease	Decrease
rs145305228	T|G	T221P	Decrease	Decrease
rs1166756826	G|C	P210R	Decrease	Increase
rs1422114933	G|A	R202W	Decrease	Increase
rs1568519097	A|T	L183Q	Decrease	Decrease
rs1323113005	C|T	A179T	Decrease	Decrease
rs763266151	T|G	T174P	Decrease	Decrease
rs758367800	A|G	F88L	Decrease	Decrease
rs145907048	A|C	F73V	Decrease	Decrease
rs775105991	C|T	R25Q	Decrease	Decrease

**Table 4:** Predict the pathogenicity and underlying functional alterations of non-synonymous SNP (nsSNPs) in AKT2 protein.

			Mut-Pred		
Variant ID	Allele	Mutation	Score	Effects	Function Affected
rs1447335111	T|C	Y475C	0.9	(−)	Loss of Relative solvent accessibility
rs1371711287	C|A	G394C	0.9	(−)	Loss of Intrinsic disorder; Loss of B-factor; Loss of Methylation at K390
rs765026585	C|A	D326Y	0.9	(−)	Loss of Relative solvent accessibility
rs930976777	G|A	R251W	0.8	(−)	Loss of Helix; Loss of Allosteric site at Y255
rs1422114933	G|A	R202W	0.6	(−)	Loss of Helix
rs1568519097	A|T	L183Q	0.9	(−)	Loss of Acetylation at K181
rs1323113005	C|T	A179T	0.9	(−)	Loss of Helix
rs763266151	T|G	T174P	0.8	(−)	Loss of Strand
rs758367800	A|G	F88L	0.8	(−)	Loss of Strand
rs145907048	A|C	F73V	0.9	(−)	Loss of Strand

(−): negative impact on protein function.

**Table 5:** Detailed summary of important non-synonymous SNP (nsSNPs) identified.

		Functional Consequence				Disease Association		Phylogenetics	Protein Stability		Function
nsSNP	Mutation	SIFT	Polyphen	SNAP2	PANTHER	SNP&GO	PhD-SNP	ConSurf	I-Mut	MUpro	Mut-Pred
rs1447335111	Y475C	*	*	*	*	**	**	5, b, average	***	***	−
rs1447335111	Y475S	*	*	*	*	**	**	4, b, average	***	***	+
rs1368133745	F470I	*	*	*	*	**	**	9, b,s, conserved	***	***	+
rs1371711287	G394C	*	*	*	*	**	**	9, e,f, conserved	***	***	−
rs749701848	P349L	*	*	*	*	**	**	9, e,f, conserved	***	***	+
rs1198321649	G346S	*	*	*	*	**	**	9, e,f, conserved	***	***	+
rs1485158704	M344K	*	*	*	*	**	**	7, b, conserved	***	***	+
rs765026585	D326Y	*	*	*	*	**	**	4, e, average	***	***	−
rs1257580103	A318V	*	*	*	*	**	**	9, b,s, conserved	***	Increase	+
rs930976777	R251W	*	*	*	*	**	**	5, e, average	***	***	−
rs753149827	R243W	*	*	*	*	**	**	5, e, average	***	***	+
rs145305228	T221P	*	*	*	*	**	**	9, e,f, conserved	***	***	+
rs1166756826	P210R	*	*	*	*	**	**	9, e,f, conserved	***	Increase	+
rs1422114933	R202W	*	*	*	*	**	**	5, e, average	***	Increase	−
rs1568519097	L183Q	*	*	*	*	**	**	6, b, conserved	***	***	−
rs1323113005	A179T	*	*	*	*	**	**	9, b,s, conserved	***	***	−
rs763266151	T174P	*	*	*	*	**	**	5, e, average	***	***	−
rs758367800	F88L	*	*	*	*	**	**	7, b, conserved	***	***	−
rs145907048	F73V	*	*	*	*	**	**	6, b, conserved	***	***	−
rs775105991	R25Q	*	*	*	*	**	**	8, b,f, conserved	***	***	+

*: deleterious; **: disease-associated; ***: decrease protein stability. e—An exposed residue according to the neural network algorithm. f—A predicted functional residue (highly conserved and exposed). b—A buried residue according to the neural network algorithm. s—A predicted structural residue (highly conserved and buried); (−) negative effects; (+) positive effects.

## Data Availability

Not applicable.
